# Service users’ and carers’ views on research towards stratified medicine in psychiatry: a qualitative study

**DOI:** 10.1186/s13104-015-1496-y

**Published:** 2015-09-28

**Authors:** Diana Rose, Constantina Papoulias, James MacCabe, Jennifer Walke

**Affiliations:** Service User Research Enterprise, Institute of Psychiatry, Psychology and Neuroscience, King’s College London, London, UK; Department of Psychosis Studies, Institute of Psychiatry, Psychology and Neuroscience, King’s College London, London, UK

**Keywords:** Service user research, Stratified medicine, Barriers to participation, Biomarkers, Antipsychotics, Schizophrenia, Qualitative methods, Focus groups, Carers

## Abstract

**Background:**

Approximately 30 % of people with a diagnosis of schizophrenia receive little to no benefit from current medications. There is therefore an urgent need to develop more precisely targeted and effective treatments. Identifying biomarkers to predict response to treatment and stratify patients into groups may be a way forward. However, we know little about service users’ and carers’ attitudes regarding such a ‘stratified medicine’ approach for psychiatry—nor how this might impact on their willingness to participate in stratified medicine research. This paper presents psychiatric service user and carer views on research to develop stratified medicine for treatment resistant schizophrenia, and explores the conditions under which people would be prepared to participate in a trial and their willingness to undergo various research procedures.

**Methods:**

Participatory methods were used throughout. A consultation was undertaken with an existing Service User Advisory Group (SUAG) in order to establish a topic guide. Service user focus groups were then conducted by service user researchers in Manchester, London and Edinburgh (totalling 18 people) and one carer focus group in London, attended by eight participants. Focus groups were digitally recorded, the transcripts analysed in NVivo 10 using a simple thematic analysis, and quotations de-identified to protect participants.

**Results:**

The data reflected enthusiasm for the potential of stratified medicine and both service users and carers demonstrated a strong desire to help others. However, some service users and carers feared poor performance on neuropsychological assessments, and reported that certain medication side effects might discourage them from undergoing procedures demanding immobility and concentration. Concerns were voiced that stratified medicine could encourage an overemphasis on biological symptoms, at the expense of psychosocial factors and subjective experience.

**Conclusions:**

People with experience of treatment resistant schizophrenia would welcome stratified medicine research; however researchers should take into account how such experience might inflect service users’ willingness to undergo various procedures in the context of this research. These results reinforce the value of service user perspectives in the development and evaluation of novel treatment approaches.

## Background

Stratified medicine is an approach to medical diagnosis that makes use of biological indicators or biomarkers to enable a division of a particular diagnostic group into subgroups (or strata) on the basis of prognosis and likelihood that they benefit from a particular treatment. Stratified medicine therefore is a step towards more tailored treatments or so-called ‘precision medicine’ [1]. There has been considerable support for this approach in physical medicine, notably oncology, where the drug Herceptin (trastuzumab) has been shown to improve outcomes for women with breast cancer, only in cases where the tumour has a particular genotype [2]. Current advances in imaging technologies and in data aggregation are making it possible to pursue the stratification of prognosis and treatment in a number of other fields, such as neurology, where the evidence base for particular treatments has been poor [3].

In this context there is growing research interest in the development of stratified medicine for psychiatry, a field where the biological underpinnings of illness are not currently well understood. Here, it is hoped that the increased diagnostic precision associated with stratification would help alleviate the considerable individual suffering and public health costs resulting from mental ill health [4]. Diagnostic precision is particularly pressing in the case of psychosis spectrum disorders: approximately 20–30 % of people with a diagnosis of schizophrenia do not respond to initial medication treatment, while in a significant proportion of cases treatment resistance persists for a number of years [5]. Furthermore, recent studies suggest that for a significant number of service users, reduction or discontinuation of medication after first episode psychosis does not lead to increase in relapse rates and may facilitate recovery [6], while a number of service users may achieve recovery through psychosocial interventions alone [7]. These findings testify to a pressing need for a stronger evidence base for treatment in this area, as the currently used symptom-based nosological categories may be bringing together heterogeneous types under a single diagnostic heading [7]. It is therefore proposed that stratified medicine research would refine this nosological picture by using biomarkers to identify homogeneous subgroups within or across present diagnostic categories. The reference to biomarkers may not be entirely accurate here, as cognitive tests—i.e. psychological markers—may prove more effective in stratifying populations in the context of psychiatric nosologies [8].

While researchers evince considerable enthusiasm for the potential of stratified medicine in psychiatry, we know little about service user levels of understanding and perceptions of stratification and of individuals’ willingness to participate in related trials. Similarly, while the literature on barriers and facilitators to participation in mental health research is currently gaining ground [9–12], evidence is scarce on how the context of stratified medicine research might impact on these. Surveys investigating public attitudes around stratification have not included mental health conditions [13, 14]. There are indications that service users may be ambivalent towards current biological accounts of mental illness and related pharmacological treatments [15] and that they may perceive the use of biomarkers in psychiatry to be stigmatising [16, 17] but this does not necessarily predict inclination to participate—or not—in stratified medicine research.

Against this background, this paper reports on a service user led qualitative study, which elicited psychiatric service users’ and carers’ perspectives on the value of stratified medicine for treatment resistant schizophrenia. This study was conducted to assist protocol design of a large stratified medicine research programme for treatment resistant schizophrenia in the United Kingdom. The programme aimed to use psychological, genetic and neuroimaging data to define biomarkers for patient populations who do not respond to currently available dopaminergic antipsychotics. The programme objective would be to test the hypothesis that such populations might show abnormalities in glutamate, rather than dopamine, pathways [18, 19] and may therefore benefit from glutamatergic medications under development. In this context, our qualitative study elicited service users’ and carers’ responses on the acceptability of various procedures involved in the proposed research programme and their views on the likelihood of participating in such a programme.

Our study thus worked to embed service user involvement in the design of stratified medicine research with the aim of improving acceptability and reach of such studies [20]. While service user involvement has gained considerable ground in health services research, its suitability for earlier phases of the ‘translational pipeline’ is contested [21]. However, there is growing evidence of the benefits of such involvement: for example service users’ experiential knowledge can improve the quality of research by introducing new, or refining existing research questions [21, 22], by increasing the likelihood of recruitment to target [23] and by improving consent processes [24]. Indeed, Callard, Rose and Wykes have recently called for substantive service user and public involvement in early phases of ‘bench to bedside’ or translational research. Such early involvement, they claim, is important because it is at the level of basic research that decisions about research priorities, funding allocations and ultimately the goals of prognosis and treatment are being shaped [25].

## Methods

### Study design

This was a two-phase qualitative study, employing participatory methods: it used an adapted version of the model developed at the Service User Research Enterprise (SURE) of the Institute of Psychiatry, Psychology and Neuroscience (IoPPN) in London. SURE conducts research from the service user perspective and employs service user researchers throughout [15]. For this study, a consultation was undertaken with an existing Service User Advisory Group (SUAG), which advises primarily, but not only, on studies at the Biomedical Research Centre for Mental Health (BRC) at the South London and Maudsley NHS Foundation Trust and the IoPPN. Secondly, focus groups were conducted by service user researchers with three sets of service users and one group of carers.

### Study sites

The consultation with the SUAG took place in London. Focus groups were held at locations in London, Manchester, and Edinburgh.

### Study population and sampling framework

Some, but not all, of the SUAG had diagnoses of treatment-resistant schizophrenia. The focus groups with service users (n = 18) took place in Mental Health Trusts in London, Manchester and Edinburgh and purposive sampling was used. All participants had a diagnosis of treatment-resistant schizophrenia but differed slightly on other parameters. The London focus group was recruited via the Mental Health Research network (MHRN) and diagnosis was the key inclusion criterion. Members of the Manchester group were living in a supported housing project and knew each other well. The Edinburgh group was recruited through the Scottish Mental Health Network (SMHRN) and comprised service users with a strong interest in research. Carers (n = 8) were recruited from a psychosis unit in a London Mental Health Trust. All were parents of people who had been treated there and they, too, were familiar with one another.

### Study period

Consultation took place with the SUAG in April 2013; data were collected from the four focus groups in May–June 2013, and analysis was conducted in late 2013 to early 2014.

### Data collection

A topic guide was constructed by two service user researchers from the Service User Research Enterprise at King’s College London (DR and CP), together with an academic clinician from the main study (JM). This guide was specifically tailored to the needs of the proposal for the larger project while also incorporating findings and suggestions from the consultation with the SUAG. The two service user researchers then facilitated focus groups using the topic guide. Focus group participants were presented with a brief account of the proposed stratified medicine programme. They were told that:

- stratified medicine meant developing clinical tests to distinguish which patients are more likely to benefit from a particular treatment;
- in the treatment of schizophrenia, this approach would involve investigating potential brain abnormalities in people who do not respond to current anti-psychotic medications;
- since these medications target dopamine pathways in the brain, people who do not respond to these may have abnormalities in different pathways;
- there are indications that glutamate pathways may be involved instead.

Written informed consent was obtained from all participants. Following this, the group discussion was structured into three topics. Firstly, facilitators invited participants’ views on stratified medicine as an approach to treatment of schizophrenia. Secondly, participants were asked about their attitudes towards procedures that might help stratify people with a diagnosis of schizophrenia. These procedures were presented in increasing levels of ‘intrusiveness’ as judged by the research team in the proposed study [neuropsychological tests, blood tests, magnetic resonance spectroscopy (MRS) and positron emission tomography (PET) scans]. Thirdly, participants were asked about their willingness to participate in a trial and their views on ways of promoting participation.

Discussions were audio recorded and transcribed with the informed consent of all participants. Transcripts were de-identified to protect the identity of participants.

### Data analysis

Two service user researchers (CP and JW) conducted a deductive thematic analysis using the topic guide [26]. Transcripts were also analysed inductively and supplementary codes identified until thematic saturation was reached [27]. These codes were then aggregated into super-ordinate themes and the coding frame was iteratively adjusted. NVivo10 software was used to store, index and retrieve textual material and to identify illustrative quotations.

### Ethical considerations

Ethical Approval was given by the NRES Committee North East—Newcastle and North Tyneside 2, reference number 13/NE/0103.

## Results

It is important to note that the focus group discussions were non-linear, and medication side-effects were an on-going theme throughout the transcripts rather than being confined to the section of the topic guide specific to this. We present central findings from each of the three topics.

### ‘Geared up for your body’: views on stratified medicine as compared to current approaches

Both service user and carer participants reported enduring a very lengthy process of ‘trial and error’ with their medication. As a result, several service users had become disillusioned about the potential of existing medications to alleviate their symptoms: they reported feeling incapacitated both by adverse effects and by the frequent experiences of withdrawal which are a part of regime change:

It takes so long for the drugs to work in your system and you’ve got to come off that one, and then put on another dose. And then it kicks in and that wouldn’t work so it’s up and down all the time. (Service user)

So when you have to go, [to] your assessments yeah? They ask how you’re feeling, well, still hearing the voices, medication don’t work apart from side effects, where do we go from here? And you’re stumped, keep trying, keep taking the medication and we’ll get back to you. They never do because there is nothing they *can* do. (Service User)

Against this shared background of disillusionment, proliferating side effects and ineffective treatments, when a particular medication proved capable of alleviating symptoms, it was greeted with particular enthusiasm and could be imbued with a life altering significance:

It made me, sort of more like me…. D’you know what I mean? I wasn’t a robot.

[It took] ten years to find, to put me on that. And when I went on Olanzapine -that had just come out- and they said you know this is a brand new drug it’s only come out, we’ll try it on you to see how it goes. So I tried that, totally different. (Service User)

And Clozapine worked but she was very sleepy […] and with great difficulty, I persuaded her psychiatrist to prescribe [Aripiprazole] for her. And that has been brilliant …. she’s now awake for a full day a lot of the time. But it has taken a long time […] at least sixteen years. (Carer)

In this context, participants were broadly enthusiastic about an approach which held open the possibility of more precisely targeted treatments for themselves or their relatives. It is worth noting that participants did not use the phrase ‘stratified medicine’ in their discussions of that approach. Instead, they noted the heterogeneity of experiences between patients sharing a diagnosis and emphasised a need for a more individualised approach, sometimes drawing analogies with other healthcare domains and using tailoring metaphors

There’s different forms of schizophrenia, not everybody’s going to have the same symptoms. But if you get given a tablet… everybody gets the same thing most of the time to start with. There’s no sort of individuality regarding the meds, but if you can get one that’s geared up for your body and how it suits your mind then yeah, I’m all for it, totally! (Service user)

It sounds sensible because the previous system is a bit like going to the opticians and handing you a box of glasses and saying ‘try them and come back in a week and see if you can see anything’ so I think it’s wonderful. (Carer)

[it would be like both of us] going to a shop, getting the exact same dress, yeah? I’d need mine a different size, erm, and we’d both need different alterations, yeah? Our bodies aren’t the same, they don’t work the same, erm, if we could make it …actually tailor make medicine for people, I think there’s only one thing you say, and that’s get on with it! (Service user)

Additionally, some participants suggested that the development of more finely honed medications may not in itself be sufficient to improve the lives of people with a diagnosis of schizophrenia. Rather, medications may form part of a more complex picture, a ‘jigsaw puzzle’, in which social contact and peer support are also paramount:

You need a holistic approach… okay, you need the meds, but you also need somebody to talk to about your illness, how it’s going to affect you, how it’s affecting other people (Service user)

You actually need lots of different things, you’re a jigsaw puzzle. […] sometimes you do need medications, sometimes you need a rest from medications because your body is screaming out, saying, no more! …sometimes I don’t want [an] antidepressant, I want a human being to sit and tell my problems to! (Service user)

### Attitudes towards, and perceived intrusiveness of different procedures

#### Neuropsychological testing

Overall, service users were familiar with neuropsychological assessments, having undergone several in the course of their treatment. For some, that familiarity meant they would agree to participate in trials involving such tests. Others were more hesitant, reporting that they had found such procedures mentally challenging:

Well you see I done one, was it last year? […] we had an interview, and then we went and done puzzles […] and I see nothing wrong with that, if it it’s gonna help, really I don’t. (Service User)

I could read the instructions and follow the instructions and sort of answer the questions. But when it came to doing like puzzles and things it was quite difficult… I can’t do those at all. (Service user)

Some carers were also hesitant, as they appeared concerned with how their relative would cope with this kind of testing, They indicated that their relatives were no longer ‘well enough’ to submit to psychological testing. Some felt that such tests demanded a higher level of cognitive and co-ordination skills than their relatives could master or tolerate—in part because of the debilitating effects of their medication.

[She] wouldn’t have been able to co-operate in tests like that at all. She wouldn’t have had the staying power or the co-ordination to do anything because of the other drugs she had been taking. (Carer)

I think the problem is with that kind of a test, I don’t know how it would go for anybody else, but my daughter when medication was first being introduced would never have been well enough to do that kind of test. You know things had got well beyond her being able to sit and, you know, make a contribution like that. It kind of needs to come earlier to have the confidence when things are first beginning to go wrong. (Carer)

#### Blood tests

Most participants regarded blood tests as routine and unthreatening, a familiar and necessary component of their or their relative’s usual treatment regime.

Well with the medication you’re actually on they have to do blood tests every so often…and I think most of us have had that many blood tests like, it’s just normal! (Service user)

My son regularly, every month, he has to have a blood test so it would be nothing new for him. (Carer)

A number of service users objected to this procedure, on the grounds of needle phobia or known physical problems with drawing blood. Despite these concerns, blood testing was more readily accepted and justified by service users than the other test types proposed.

I’m scared of needles but if it would help my mind, I would yeah, but the other one [PET scan] sounds like needles in both arms and drips and it lasts for a few hours. (Service user)

I’m not particularly happy with them because I haven’t got any veins and it’s very difficult… A bit too intrusive and they hurt. (Service user)

#### Magnetic resonance spectroscopy (MRS)

The testing duration and enclosed space of an MRS scanner proved more challenging for both service users and carers, who spoke of having difficulty sitting still and experiencing anxiety around small spaces. Here again, carers appeared concerned that their relatives may not be well enough to undergo such procedures:

The only issue I’ve got with that is sitting in a machine for two to three hours… that’s a hell of a long time. (Service user)

Being big in them means I feel trapped if it had been an accident or something went wrong with the machine I’d feel that, to get my bulk out of that machine and get everything that the nurse is telling me would just be so uncomfortable. (Service user)

I think though that with a scan like that, the person would have to be quite well to undergo it. I think that somebody who was having anxiety or anything like that would find it extremely difficult to be put into a tube and I think that would be very difficult. (Carer)

Additionally, some carers suggested that a scanner that is too small may be psychologically challenging to people who have experienced considerable weight gain through treatment:

I could see that [relative’s name] … when she was very big, would’ve felt uncomfortable going in there and if she’d not fitted; it would’ve been something else that would’ve not been good for her, to have not fitted in the tube. (Carer)

#### Positron emission tomography (PET) scans

All participants considered PET scans to be a particularly intrusive investigation, with their inherent demands on time and patience compounded by fear of needles and radioactive tracers.

You’re still talking about 2-3 h of being pricked and prodded and cameras clicking on you. It’s just a nightmare. You wouldn’t expect that under normal circumstances… So the thought of lying dormant for two to three hours, while people are taking blood and giving you radioactive doses of whatever, and being scanned at the same time, that’s hard work. They’d [be] better off sedating you and doing it. (Service user)

However, this stance was countered by one service user, who believed that the intrusiveness of a procedure was a sign of its rigour and scientific authority, and therefore of its potential usefulness

It sounds rather too extreme. And usually for medicine I think if things are that extreme, that means it going somewhere and it makes it easier to find out or to get rid of or find out what they’re trying to find out about my, my mental state so… it must be a hopeful thing to do. (Service user)

Again, some carers were doubtful whether their family member would be well enough to undergo this process.

I think he’d sit still for that time but whether he’d consent to it once again is difficult to say…one day he’s a different person to another. (Carer)

### Willingness to participate in a trial and suggestions for promoting participation

Despite the aforementioned concerns, when participants were explicitly asked whether they or their relatives would choose to take part in trials, most confirmed that they would. These participants were largely motivated by the hope that others would not have to endure distressing and ineffective treatments in the future:

I would take part. Despite the inconvenience […] I would take part, coz I would see it as something – I’d obviously not benefit from it now – it would help other people in the future. And so long as there’s not gonna be any harm to myself by doing it, I would agree to take part (Service user)

… I would do it in the respect that it’s gonna help people in the future, [not to go] through the processes that we’ve been through; I would do it for that. (Service User)

I think at the moment she’s so well that I don’t think it would unbalance her and knowing, and she’s always been very keen on medical things and things like that, I think she would want to help because she would feel that she was helping other people by doing this. (Carer)

However, those service users and carers who, after such distressing and ineffective treatments, had found one that worked for them or for their relative, were more reluctant to take part in procedures which, they feared, might compromise their hard won equilibrium.

… if I definitely had to go off my meds I’m on just now, I’d be scared of what was to happen when I came off them, because it’s taken so long to get on at last, to get everything just perfect. You know what I mean? (Service user)

I would like to think she’d do perfectly well and not bother, but is it worth taking the risk? I mean she’s been so unwell in the past, she’s so well now, why on earth interfere with any of it? (Carer)

In this context, receiving clear information about the purpose of the trial and any attendant risks, as well as feeling that participants’ concerns were respected, became particularly important. A desire for (medical or peer-led) reassurance about the procedures and their outcomes was a common theme.

I’d probably want to speak to a physician before going inside one of those things but as long as there’s preliminary need for me then I think it would be okay. (Service user)

If they were fully informed and they realise that there were no consequences from it. For example that if you’re going to inject radioactive dye, that there are absolutely no consequences from that…And also I think they would need reassurance that what they’re doing is actually going to benefit other people. (Carer)

Other participants focused on the need to alleviate boredom or withdrawal symptoms throughout the more involved testing procedures.

I’d have to listen to music, I mean I’d have to take that with me and just sit… I couldn’t see myself sitting there with nothing for two to three hours like ‘cos I get fidgety. (Service user)

Just give me something I can take, put a patch on, or nicotine chewing gum! (Service user)

## Discussion

Stratified approaches have clear implications for improving quality of life and treatment adherence in (what is currently regarded as) ‘drug-resistant’ schizophrenia. In our study we presented stratified medicine in psychiatry as a way of determining which sub-groups of people are more likely to respond to a specific treatment. We thereby indicated that such a development would potentially curtail lengthy and ineffective treatments. In this context, our participants’ enthusiasm is not surprising. While participants did not adopt the proposed terminology, they clearly understood that what was being proposed was a treatment more closely tailored to individual bodily needs. Participants felt that pursuing this goal would be a welcome development, insofar as it promised to give the field of psychiatry a scientific precision that is currently lacking. What is more significant for the design of studies and the development of recruitment strategies in this field is how we may identify and attend to the particular anxieties which may accompany this apparent willingness to participate in trials.

Participants voiced a number of concerns about the different test types required for such studies: of these, worries relating to lengthy or invasive procedures and confined spaces aligned with researchers’ judgements (in ordering the procedures in the focus groups) about which methods would be interpreted as most intrusive. In discussing participants’ tolerance for different procedures, we had anticipated that service users and carers would find PET scans considerably more challenging and intrusive than neuropsychological tests. This is because the former involve physical discomfort, a considerable time commitment and the administration of radioactive tracers. However, participants’ responses suggested that, in some cases, neuropsychological tests might also be perceived as intrusive, insofar as engaging with challenging mental tasks could be seen as destabilising. It is also possible that some carers’ reluctance towards neuropsychological tests stemmed from a perception that their relatives would be evaluated during such testing, and a concern that a poor result on the test (partly consequent to medication side-effects) might be deleterious for the individual’s self-esteem. This finding has considerable bearing on stratification research, particularly so since, in psychiatry, by contrast to other clinical fields, neuropsychological tests are expected to play a central role in stratification [8]. Similar remarks about the effect of procedures on participants’ self-worth were voiced by some carers in relation to scans: they argued that those of their relatives who have become overweight in the course of treatments might find entering the constricting space of an MRS scanner not only physically challenging, but also potentially humiliating.

Taken together, these findings present us with two potential barriers to participation in stratification trials. These barriers may be particularly pronounced for people with a diagnosis of treatment resistant schizophrenia who have experienced years of ineffective treatments. Firstly, researchers will need to take into account the impact of the likely adverse effects associated with lengthy use of anti-psychotic medications: some of these side-effects (in particular weight gain, poor concentration, akathisia and incontinence) may make participants reluctant to undergo procedures which will involve constricted spaces and immobility. Secondly, some of these procedures might be perceived to be mentally rather than physically challenging and thus potentially compromising to service users’ sense of self-worth. While the latter finding is associated mostly with carers who may have been over protective of their relatives, it is nevertheless important to be mindful of the potential effect of procedures on participants’ self-esteem and sense of dignity.^1^

Additionally, our findings suggest that there might be reluctance to participate in stratified medicine from (1) people who have—after many years—found an anti-psychotic medication that they feel is right for them; (2) people who are concerned that a focus on biomarkers precludes adequate research and therapeutic attention to psychosocial factors. Further research would be needed to probe these concerns in greater detail.

Consequently, an understanding of the history and experiences of people with treatment resistant schizophrenia, of the impact of this history on service users’ concerns about undergoing potentially taxing procedures, and on their attitudes on the value and effectiveness of medical interventions should be an important factor in study design. To this end, early and substantive involvement of service users and carers in stratified medicine trials can greatly enhance such understanding and therefore expedite the progress of research in this field.

### Limitations

The results of the present study are restricted by a lack of demographic data on focus group participants, meaning it is not yet possible to examine whether gender, age, or other background factors shape personal opinion on stratified medicine or engagement with research. Furthermore, it is important to remember that intention (e.g. to participate in stratified medicine research, or to imagine that a relative would participate in stratified medicine research) cannot predict actual behaviour. Additionally, the topic guide did not invite participants’ views on the possible uses of samples after testing; this topic featured more strongly in a recent UK public survey of views on stratification in general medicine [13]. At the same time, no participant in the present study spontaneously mentioned this. In a qualitative study of trial participants’ understanding of pharmacogenetic medicine, service users expressed no worries that their blood samples would be held centrally [28]. One may speculate that mental health service users are accustomed to information about them being shared, or that sharing of records is conceptualised differently from the sharing of biological material. However, caution should be exercised in generalising from these results, as research on psychiatric patients’ views on this topic is scarce; potentially a double-edged sword in an era where ‘big data’ are becoming more important.

Finally, it is important to note that questions of aetiology did not figure prominently in our study. Since the study was preparatory to a large trial, its purpose was instrumental (to elicit service user and carer views on research on stratification, in order to refine protocol design for a trial) rather than exploratory (to explore service user and carer perceptions of stratification in psychiatry).

## Conclusion

Our findings suggest that both service users and carers show considerable enthusiasm for stratified approaches in the treatment of schizophrenia. Despite considerable anxieties about potentially invasive procedures, we can reasonably expect this enthusiasm to translate into willingness to participate in trials, as altruistic motivation is strong in this area. The provision of clear information, willingness to engage with service user needs and, potentially, peer support mechanisms will likely enhance recruitment to trials in this area. Therefore, collaborating with service users in the early stages of study design—for example, to understand in relation to which particular research procedures participant anxieties or concerns are likely to be greater—would be beneficial and may increase the likelihood of recruitment to target.

## Footnotes

1 It is important to note here that our findings resulted in an alteration to the protocol of the stratified medicine study: the neuropsychological tests chosen were the briefest possible in order to reduce their potential burden on participants.
